# Self-evolving experimental platform for 3D sand printing

**DOI:** 10.1093/nsr/nwag352

**Published:** 2026-06-09

**Authors:** Songtao Hu, Xiantong Zhang, Kaiming Tang, Haoran Li, Wenhui Lu, Zipeng Wang, Yinjun Deng, Bo Zhang, Xiaobao Cao, Xi Shi, Zhike Peng

**Affiliations:** State Key Laboratory of Mechanical System and Vibration, School of Mechanical Engineering, Shanghai Jiao Tong University, Shanghai 200240, China; State Key Laboratory of Mechanical System and Vibration, School of Mechanical Engineering, Shanghai Jiao Tong University, Shanghai 200240, China; State Key Laboratory of Mechanical System and Vibration, School of Mechanical Engineering, Shanghai Jiao Tong University, Shanghai 200240, China; School of Mechanical Engineering, Ningxia University, Yinchuan 750021, China; State Key Laboratory of Mechanical System and Vibration, School of Mechanical Engineering, Shanghai Jiao Tong University, Shanghai 200240, China; State Key Laboratory of Mechanical System and Vibration, School of Mechanical Engineering, Shanghai Jiao Tong University, Shanghai 200240, China; State Key Laboratory of Mechanical System and Vibration, School of Mechanical Engineering, Shanghai Jiao Tong University, Shanghai 200240, China; State Key Laboratory of Mechanical System and Vibration, School of Mechanical Engineering, Shanghai Jiao Tong University, Shanghai 200240, China; School of Mechanical Engineering, Ningxia University, Yinchuan 750021, China; Guangzhou National Laboratory, Guangzhou 510320, China; State Key Laboratory of Mechanical System and Vibration, School of Mechanical Engineering, Shanghai Jiao Tong University, Shanghai 200240, China; State Key Laboratory of Mechanical System and Vibration, School of Mechanical Engineering, Shanghai Jiao Tong University, Shanghai 200240, China; School of Mechanical Engineering, Ningxia University, Yinchuan 750021, China

**Keywords:** sand printing, casting, automated experimentation, artificial intelligence

## Abstract

Three-dimensional (3D) sand printing revolutionizes casting mold fabrication while confronting nonlinear high-dimensional optimization challenges stemming from variable combinations and objective conflicts. Here, we develop Ex3D sand printing (Ex3DSP), a self-evolving experimental platform combining robotic ‘can-do’ capabilities with artificial intelligence ‘can-think’ cognition to establish a‘do-while-thinking’ self-evolving experimentation paradigm. Addressing a three-objective, three-variable high-dimensional optimization problem, our Ex3DSP discovers the Pareto front with a 1148-fold reduction in experimental workload. The platform provides two solution types (objective-optimal and balanced) with interpretability and reveals underlying mechanisms (variable-objective interaction and sand mold fracture behavior). Moreover, these solutions are applied to guide real-world casting parameter selection, yielding castings with up to 385% improved physicochemical and mechanical performances. This work demonstrates a self-evolving experimentation paradigm for 3D sand printing, offering both an intelligent additive manufacturing roadmap and pre-industrial screening potential.

## Teaser

Self-evolving experimental platform revolutionizes 3D sand printing, enabling “do-while-thinking” precise discovery of high-performance processes.

## INTRODUCTION

Droplet-based 3D printing has emerged as a pivotal additive manufacturing technology across diverse fields, including precision casting [1,2], lightweighting/complex components [3,4], flexible electronics [5,6], and biomedical applications [7,8]. Among these, 3D sand printing (3DSP) is revolutionizing foundry practices by overcoming the inherent constraints of conventional pattern-based methods through digital process flexibility, tooling-free fabrication, and complex geometry enablement [9,10]. However, multivariate process parameters critically determine the casting performances of printed sand molds, encompassing droplet-related [11,12] (e.g. binder composition, volume, and velocity) and sand-related [2,13] (e.g. sand composition, morphology, and layer thickness; curing agent composition and proportion) attributes. Such variable combinations engender a vast parameter space, transforming 3DSP optimization into a high-dimensional challenge. Moreover, the conflicts between objectives (e.g. mechanical strength [14,15], air permeability [15,16], and gas generation [16,17]) substantially complicate trade-off analysis, further intensifying the challenge in high-dimensional optimization.

Notably, this high-dimensional challenge in 3DSP, stemming from variable combinations and objective conflicts, exhibits strong nonlinearity owing to cross-coupled interactions and coexisting synergy-antagonism effects in variable-objective relationships. Although numerical simulations can model the wetting and penetration of droplets in sand [18,19], the stochastic nature of droplet-powder interactions and post-processing phase transitions render computational approaches ineffective for high-dimensional optimization, leaving experimental investigation as the only viable pathway. However, a vast variable space (e.g. given three variables, discretized in percentages at intervals of 2.5% requires evaluating 68 921 potential combinations) renders traditional trial-and-error experimentation fundamentally impractical, as it is intrinsically limited by time- and cost-intensive exploration and batch-effect-induced variability, restricting experimental investigation to small-scale, suboptimal solutions. Gratifyingly, the integration of automation and artificial intelligence (AI) offers a feasible solution that merges execution (‘can-do’) and reasoning (‘can-think’) capabilities into a self-evolving (‘do-while-thinking’) cycle. By this, high-throughput experimental platforms reliably generate standardized data, while AI-driven analytics dynamically refine experimental strategies by decoding underlying relationships and balancing objective trade-offs, drastically reducing experimental iterations.

Recent advances have demonstrated self-evolving experiment validation [20], also termed AI-driven [21–24], AI-accelerated [25], self-driven [26–30], autonomous [31,32], self-optimizing experiments [33], and even robotic scientists [34,35]. These works optimized diverse material properties, including strength [28,36], elongation [36], conductivity [37,38], energy density [24], and biological adsorption [20]. Besides addressing variable combinations, several studies have specifically highlighted the feasibility of self-evolving experiments for addressing objective conflicts, exemplified by strength versus specific volume in bioinspired composites [39], and conductivity versus processing temperature in palladium films [38]. However, no implementations of self-evolving experiments have been reported for 3DSP, including both single- and multi-objective high-dimensional optimization scenarios.

**Figure 1.**
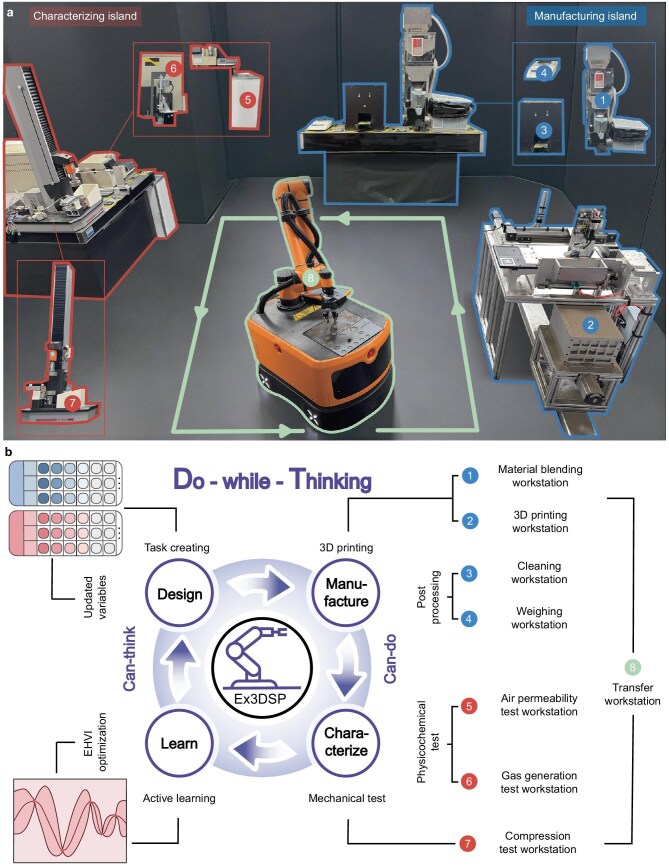
Self-evolving experimental platform Ex3DSP. (a) The Ex3DSP is composed of a robotic platform (2 functional islands comprising 8 automation workstations) and a multi-objective active learning framework. (b) Self-evolving experimentation paradigm for 3D sand printing, incorporating the design-manufacture-characterize-learn cycles.

Here, we report a self-evolving experimental platform, Ex3DSP (Fig. 1), for casting performance optimization of sand molds, which integrates a robotic body (two functional islands comprising eight self-developed/modified automation workstations for standardized experimental data generation to achieve ‘can-do’ capability) with a multi-objective active learning brain (directionally tuning experimental protocols to ensure ‘can-think’ capability), constructing a ‘do-while-thinking’ self-evolving experimentation paradigm. For pedagogical demonstration, a high-dimensional optimization problem for three key objectives (casting performances of sand molds, including air permeability AP, gas generation GG, and compressive strength CS) against three main variables (process parameters of 3D printing, including nozzle voltage NV, layer thickness of sand LT, and curing agent proportion in sand CAP) is posed to the Ex3DSP. The Ex3DSP successfully discovers the Pareto front (PF) after 60 combinations, achieving a 1148-fold reduction in experiment workload compared to exhaustive evaluation of 68 921 potential combinations. The PF provides two types of optimal solutions (objective-optimal type with their dominated performance maximized at the expense of other performances; and balanced type across all performances) with interpretability (exploratory search, backbone construction, convergence filtering, and marginal exploitation). Interestingly, variable-objective interaction mechanisms (e.g. NV-CAP ratio dominance) and sand mold fracture mechanisms (e.g. strength-embrittlement trade-off) are elucidated. Moreover, the optimal solutions are adopted to guide process parameter selection for real-world casting applications, achieving castings with enhanced physicochemical and mechanical performances (e.g. 385% relative improvement in tensile strength). We believe this study to be the very demonstration of a self-evolving experimentation paradigm for 3D sand printing, providing an intelligent roadmap for additive manufacturing and offering potential applications in primary screening prior to industrial intensification.

## RESULTS AND DISCUSSION

### Self-evolving experimental platform

Our Ex3DSP integrated a robotic platform (two functional islands comprising eight self-developed/modified automation workstations) and a multi-objective active learning framework (Fig. 1a), iteratively executing design-manufacture-characterize-learn cycles without human intervention to construct a ‘do-while-thinking’ self-evolving experimentation paradigm for 3D sand printing (Fig. 1b). The two islands served as higher-level units coordinating the subordinate workstations, with one dedicated to specimen manufacturing (in blue) and the other to property characterization (in red). In designing, process parameters were recommended to create experimental tasks. In manufacturing, sands and curing agents were proportionally mixed in a self-developed material blending workstation, then liquid binder was jetted from the printhead in a self-developed 3D printing workstation to consolidate the mixed layers into sand molds, before sequential transfer to self-developed cleaning workstation and modified weighing workstations for post-processing. Herein, the 3D printing workstation executed powder spreading, surface leveling, binder jetting, chemical curing, and layer stacking manufacturing sequences to fabricate sand molds (Fig. S1). In characterizing, tests for air permeability, gas generation, and compression strength were subsequently performed in three modified workstations for physicochemical and mechanical objective acquisition, which were automatically extracted and converted via a customized data analysis pipeline to ensure accuracy and consistency. Detailed protocols are provided in the section Methods. In learning, the resulting experimental data were used to train a Gaussian Process Regression (GPR) surrogate model, supporting an Expected Hypervolume Improvement (EHVI) criterion in recommending optimal values for the next design step. Besides, a mobile transfer workstation equipped with a six-axis robotic arm, electric gripper, and depth camera autonomously managed raw material supply and specimen localization/grasping/transportation, ensuring efficient interconnection and seamless integration across all workstations. To minimize scale-up effects as much as possible, Ex3DSP adopted the same key material system used in industrial sand printing, and the optimized parameter ranges were selected to remain relevant to industrial practice. More information about the Ex3DSP is provided in the Methods section, Figs S2–S4, and Video S1.

### Pareto front discovery

**Figure 2.**
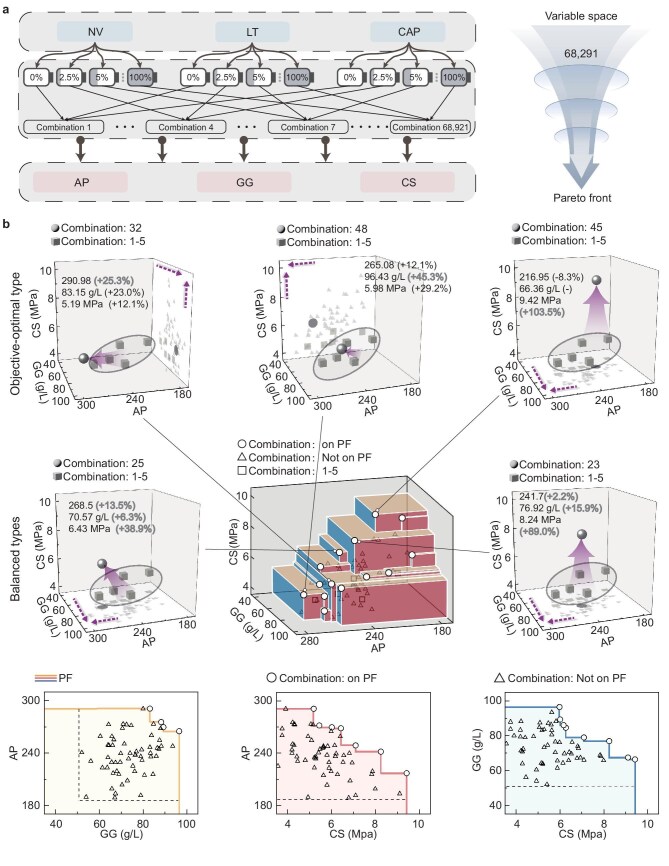
Establishment of the Pareto front. (a) High-dimensional optimization problem with 3 objectives and 3 variables. (b) 3D and 2D Pareto front with 14 solutions, including objective-optimal and balanced types.

For pedagogical demonstration, a high-dimensional optimization problem for three key objectives (casting performances of sand molds, including AP, GG, and CS) against three main variables (process parameters of 3D printing, including NV, LT, and CAP) was posed to the Ex3DSP (Figs 2a and S2a). AP, GG, and CS were selected as objectives because they are widely recognized as the key casting performance indicators for sand molds by affecting defect formation and structural reliability (Table S1) [14, 40, 41]. Correspondingly, NV (positively correlated with binder droplet size), LT, and CAP were selected due to their governing roles in the above objectives. To discover sand molds with superior casting performances, an exhaustive set of variable combinations must be considered to guarantee sufficient exploration breadth. Sampling each process parameter at 2.5% intervals across the full range yielded a high-dimensional variable space of 68 921 potential combinations. Of note, each combination required multiple tests for multi-objective optimization, coupled with replicated fabrication to ensure testing reliability and parallelism, further enlarging the practical exploration space. Specifically, the Ex3DSP conducted five GG specimens, three AP specimens, and three CS specimens for each combination.

Regarding such multi-objective high-dimensional optimization, automation endowed only with ‘can-do’ capability is insufficient. The Ex3DSP harnessed its ‘can-think’ capability to rapidly converge toward the PF boundary through a self-evolving paradigm. Specifically, it employed a multi-objective active learning framework (Fig. S2b), through which experiments were adaptively steered toward high-value regions of the variable space, thereby avoiding the inefficiency of random search or exhaustive enumeration. The framework operated in a six-stage loop. In Stage I, candidate variable sets (V1, V2, V3) and their corresponding objectives (O1, O2, O3) were incorporated into the training dataset and normalized. In Stage II, the GPR surrogate model was trained for each objective to capture predictive distributions [36,38]. In Stage III, candidate combinations were sampled from the variable space, and their objectives were predicted and evaluated by the surrogate model to construct a pool of potential solutions. In Stage IV, the EHVI was employed as the criterion to quantify each candidate’s potential contribution to the expansion of the current PF [42,43]. In Stage V, the top-ranked candidate was selected for 3D printing and performance testing. Each observation either expanded the Pareto front or improved the model’s local accuracy for subsequent iterations. In Stage VI, the experimental results were appended to the dataset to update the surrogate model, initiating the next iteration. Of note, a defining feature of the above framework is its self-cycling mechanism: each experimental outcome is immediately fed back to the surrogate model, with hypervolume serving as a global quality indicator that drives progressive convergence toward the true PF [44]. Unlike approaches that rely on single-objective or local criteria, hypervolume integrates the overall quality of the solution set, thereby guiding the search toward both improved solutions and broader coverage of the objective space. Further methodological details (e.g. description of GPR and EHVI) are provided in Methods and Note S1.

Starting from 5 orthogonal combinations, the Ex3DSP executed 55 combinations (60 in total) to successfully discover the 3D PF comprising 14 PF solutions (Fig. 2b; error bar version is provided in Fig. S5). These 14 PF solutions can be categorized into two types (taking five representatives): objective-optimal and balanced. The objective-optimal representatives included AP-optimal (Combination 32), GG-optimal (Combination 48), and CS-optimal (Combination 45) solutions. Compared with the average performance of initial combinations, the AP-optimal (25.3%, 23.0%, and 12.1% in AP, GG, and CS) and GG-optimal (12.1%, 45.3%, and 29.2% in AP, GG, and CS) solutions improved all three objectives simultaneously, with their dominant targets maximized. In contrast, the CS-optimal solution exhibited trade-offs, achieving a remarkable 103.5% gain in CS while maintaining GG but compromising AP by 8.3%. Regarding balanced solutions, they demonstrated overall favorable performances without extreme trade-offs, delivering improvements of 13.5%, 6.3%, and 38.9% (Combination 25) and 2.2%, 15.9%, and 89.0% (Combination 23) across AP, GG, and CS. Notably, the identified PF was established through broad exploration of the three-variable space rather than a narrowly confined local search (Fig. S6). Additional perturbation experiments around two representative Pareto solutions (Combinations 23 and 45) further confirmed their near-optimality within the neighboring parameter space (Table S2).

The 3D PF revealed the intrinsic trade-offs among the three objectives. The distribution of solutions showed that improving CS was accompanied by reductions in both AP and GG, whereas AP and GG could be improved concurrently over a broad range but at the expense of CS. The trade-offs existed between CS and the other two, while AP and GG were relatively cooperative. To better disentangle these relationships, the 3D PF was projected onto 2D planes to provide a clearer view of the pairwise trade-offs. The projections again highlighted the above relationships: the PF in the AP-GG plane remained comparatively flat, with only a few extreme high-value points showing limited trade-offs, the PFs in the CS-AP and CS-GG planes exhibited steep descending fronts, reflecting pronounced trade-offs. Notably, compared with the 2D projections, the larger number of PF solutions observed in 3D underscored that the trade-offs intensify substantially when all three objectives are optimized simultaneously.

It should be noted that the PF established here was based on a specific ceramic sand-binder-curing agent system. Despite this, the selected ranges of NV, LT, and CAP are compatible with industrial sand mold printing equipment. For other sand-binder-curing agent systems, involving different sand types (e.g. ceramic sand, silica sand, chromite sand, or zircon sand), binder types (e.g. furan resin, phenolic resin, or inorganic binder systems), or curing-agent types (e.g. sulfonic acid-based or phosphoric acid-based systems), the exact optimal parameter combinations may need to be re-identified by redefining the parameter ranges and reconstructing the PF on the proposed platform.

### Self-evolving process and mechanism

**Figure 3.**
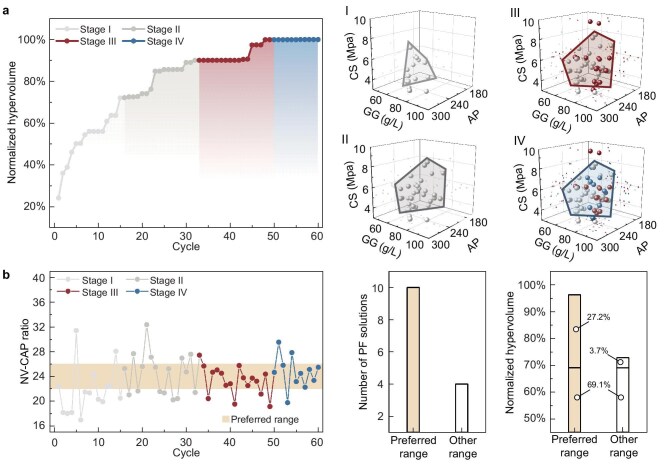
Self-evolving process and mechanism. (a) Hypervolume during the self-evolving discovery in objective space. (b) NV-CAP ratio as a representative variable configuration in variable space.

To detail the self-evolving process of the above PF discovery and examine the underlying mechanisms, from the perspective of objective space, the hypervolume (normalized by the min-max method for comparability) was employed as a function of design-manufacture-characterize-learn cycles (Fig. 3a, and the raw data are provided in Table S3). Initiated from five orthogonal combinations, the hypervolume achieved convergence within another 55 combinations (60 in total), indicating a 1148-fold reduction in experimental workload compared to the exhaustive evaluation of 68 921 potential combinations. The convergence criterion was defined as 15 consecutive iterations with hypervolume increments below 3%, where each increment was calculated relative to the hypervolume of the previous iteration. A threshold sensitivity analysis of the convergence threshold was performed using alternative values of 5%, 2%, and 1% (Fig. S7). Selecting a 5% threshold resulted in premature termination at the 33rd iteration and altered the final PF. In contrast, thresholds of 2% and 1% both led to convergence at the 57th iteration and yielded an unchanged PF. These findings support the reasonableness of using 3% as the convergence threshold in this study. The trajectory can be clearly divided into four stages. In Stage I (exploratory search), the hypervolume rose sharply as early trials rapidly identified feasible PFs; in Stage II (backbone construction), as the PF backbone gradually emerged, the hypervolume showed continued but slower expansion; in Stage III (convergence filtering), some inferior PFs were eliminated with the boundary stabilized, leaving the hypervolume at a plateau; and in Stage IV (marginal exploitation), newly identified PFs mainly filled residual gaps, making the hypervolume improve by only 0.06%. Interestingly, the PF initially expanded primarily along the CS axis, reflecting the strong trade-off between CS and GG. Once that direction approached its boundary and hypervolume gains diminished, the PF evolution shifted toward refinement within the AP-GG plane. Owing to the relatively mild conflict between AP and GG, later-stage improvements mainly manifested as frontier densification and completion, contributing little to further hypervolume expansion, while the inherent trade-offs among the three objectives became increasingly evident. Further, to compare the performance of different surrogate-modeling algorithms, multiple in silico virtual experiments were performed (see Note S2, Figs S8 and S9 for details).

Besides the objective space, the variable space provides another equally important perspective for understanding how the self-evolving process learns variable interdependence and develops preferential exploration directions. Among the three variables, the NV-CAP ratio emerged as a representative variable configuration, reflecting the intrinsic coupling between binder droplets and curing agent that governs the curing reaction. Throughout the self-evolving process, sampling showed pronounced concentration within a preferred NV-CAP range of 22–26 (Fig. 3b, left). By comparison, LT operated at a more macroscopic process level. Ratios involving LT (NV-LT or CAP-LT) showed neither clear mechanistic relevance nor stable trends (Fig. S10). When examining the distribution of the 14 discovered PF solutions across NV-CAP ratios (Fig. 3b, middle), the preferred range contained 10 PF solutions, ∼2.5 times more than other ranges. Regarding the normalized hypervolume, beyond the 69.1% shared by both regions, the preferred range accounted for an additional 27.2% of total hypervolume versus just 3.7% from the other ranges (Fig. 3b, right, the shared hypervolume is introduced in Note S1). Collectively, these findings from both objective and variable spaces demonstrate the self-evolving process’s interpretability beyond black-box optimization.

### Variable-objective interaction mechanism

**Figure 4.**
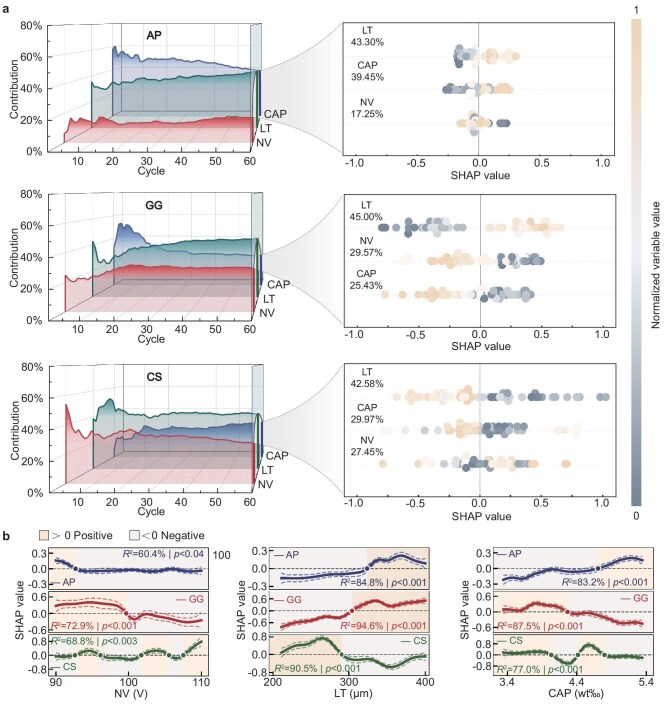
Variable-objective interaction mechanism. (a) SHAP analysis during the self-evolving discovery visualized in waterfall plots (left), with the distribution of Shapley values visualized in beeswarm plots (right). (b) Continuous variable-objective relationships visualized in generalized additive models.

Despite revealing some coupling rules (e.g. NV-CAP ratio), the variable-objective interaction mechanisms still require systematic quantification. We employed Shapley Additive exPlanations (SHAP), a game-theoretic and interpretable feature attribution approach that deciphers the black-box nature of AI machine learning [37,45], to quantify variable importance against objectives (Fig. 4a). The waterfall plots (left) illustrate the evolution of contributions from NV, LT, and CAP to AP, GG, and CS, with each contribution calculated as the mean absolute SHAP value averaged over all combinations per cycle. As the self-evolving discovery progressed, variable contribution fluctuations gradually diminished and eventually stabilized, indicating that the Ex3DSP effectively captured consistent variable-objective relationships. The final dataset’s SHAP values were aggregated into beeswarm plots (right), where variables are influence-ranked, with the horizontal axis showing effect magnitude/direction and the vertical width indicating combination clustering density. Color represents normalized variable values (gray: lower, yellow: higher). Among the variables, LT showed the highest importance across all objectives, as its regulation operated at a more macroscopic level than NV and CAP by simultaneously affecting binder droplet infiltration/dispersion and their interaction with the curing agent. With proper LT control, GG and CS exhibited nearly equal sensitivity to NV and CAP (∼30% each), confirming the NV-CAP ratio’s decisive optimization role. Conversely, AP was primarily governed by LT and CAP (∼40% each), while NV contributed only ∼17%. This distribution indicates that the NV-CAP ratio critically influences GG and CS while minimally affecting AP. These variable-objective interactions can also be understood from physicochemical perspectives, with NV mainly affecting droplet saturation and binder bridge formation, LT mainly governing pore structure and interlayer compactness at a more macroscopic level, and CAP mainly regulating curing behavior and bonded-structure integrity (see Note S3 for details).

While beeswarm plots show SHAP value distributions across combinations, they do not clearly reflect continuous variable effect variations. Therefore, we smoothed the final dataset’s SHAP values to construct generalized additive models (GAM) (Fig. 4b), enabling clearer visualization of continuous variable-objective relationships [46]. In these curves, the horizontal axis represents variable values and the vertical axis shows corresponding SHAP values, with yellow indicating positive contributions and gray indicating negative contributions. A gray-to-yellow transition reflects positive correlation, while yellow-to-gray indicates negative correlation; the yellow-gray boundary marks the threshold where a variable’s effect switches between promoting and inhibiting. Specifically, for AP, both CAP and LT showed positive correlations while NV was negatively correlated. For GG, LT displayed a strong positive correlation, whereas NV and CAP were negatively correlated. For CS, NV showed a positive correlation while LT and CAP exhibited negative correlations. Furthermore, the GAM curves revealed nonlinear characteristics through inflection points (where variable influence strength changes, indicating response sensitivity transitions) and saturation regions (flattened curve segments indicating response saturation or process stabilization). In addition, partial reversals (curve bending returning toward previous trends) reflected competitive variable influences and nonlinear compensation effects. Of note, for CS, both NV and CAP curves showed localized nonlinear variations as partial reversals, arising from LT’s dominant influence combined with NV-CAP nonlinear coupling (Tables S4 and S5). In a limited high-NV interval, the SHAP contribution of NV to CS showed a slightly negative value, which can be physicochemically understood as the combined effect of higher LT, weakening interlayer bonding and reducing local compactness, and sufficiently high CAP, causing excessive curing and damaging the mechanical integrity of the bonded structure. Detailed SHAP and GAM analyses methodology is provided in Note S4.

### Sand mold fracture mechanism

**Figure 5.**
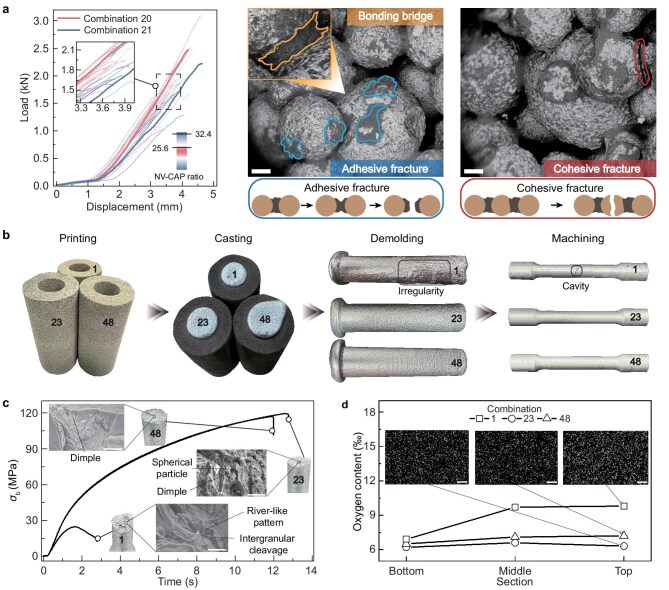
Sand mold fracture mechanism and casting applications. (a) Strength–embrittlement trade-off observed in the compression tests and the corresponding fracture modes: adhesive and cohesive. (b) Aluminum alloy castings. (c) Tensile testing and SEM images for castings after machining. (d) Oxygen elemental mapping obtained by EDS for castings after demolding. Scale bar: 50 μm.

During the self-evolving discovery, all sand molds exhibited instantaneous shear-dominated fracture in the compression tests (i.e. CS), though pre-fracture deformation varied significantly across different variable combinations. Comparing two representative combinations with nearly identical LT: Combination 20 (NV = 94.3 V, LT = 297 μm, CAP = 3.69 wt‰, NV-CAP ratio = 25.56) showed 11% higher compressive strength but fractured at 10.5% shorter displacement than Combination 21 (NV = 108.2 V, LT = 293 μm, CAP = 3.34 wt‰, NV-CAP ratio = 32.40), indicating a strength-embrittlement trade-off where enhanced load-bearing capacity reduces deformability. The corresponding scanning electron microscopy (SEM) examination revealed sand particles interconnected by bonding bridges formed during curing (in yellow), exhibiting two fracture modes during compressing (Fig. 5a). Adhesive fracture (in blue) occurred through bridges rupture at particle interfaces, showing pronounced necking before complete separation, explaining longer displacement and lower strength in Combination 21. Cohesive fracture (in red) maintained intact bridges with fracture propagating through particles themselves, explaining shorter displacement and higher strength in Combination 20. The introduction of advanced measuring techniques (e.g. X-ray, nano-/micro-indentation, *in situ* SEM) would facilitate a more comprehensive understanding of the fracture mechanism.

### Casting application

Through the self-evolution discovery, the posed three-objective, three-variable high-dimensional optimization problem was resolved by the Ex3DSP. Here, we implemented optimized combinations to guide process parameter selection for real-world casting applications (Fig. S11), selecting two representative combinations from the established 3D PF (Fig. 2b): objective-optimal Combination 48 and balanced Combination 23, with Combination 1 as reference. The two optimized combinations exhibited complementary enhancement exceeding 20% in AP/GG/CS versus the initial combination. Three hollow cylindrical sand molds [2] (length: 4 in, inner diameter: 1 in, wall thickness: 0.5 in) were printed using these combinations (Fig. 5b). After pouring ZAlSi12 aluminum alloy and demolding, castings were machined into standard tensile specimens conforming to ASTM E8-13a for uniaxial tensile testing [47]. The optimized castings displayed defect-free surfaces post-demolding, while the initial casting showed surface irregularities and internal cavities. These defects resulted from both poor AP and GG (causing severe gas entrapment during solidification) and insufficient CS (leading to surface collapse upon molten aluminum contact, Fig. S12), highlighting the practical significance of simultaneously optimizing all three performances for casting applications.

The resulting castings were subjected to uniaxial tensile testing to determine tensile strength *σ*_b_ with fracture morphologies subsequently examined via SEM (Fig. 5c and Fig. S13). The initial casting fractured after 3-second loading, achieving *σ*_b_ = 24.60 MPa—far below ZAlSi12 aluminum alloy’s intrinsic strength. Both optimized castings reached ∼120 MPa, representing 385% relative improvement while meeting application requirements, with a high degree of consistency. The initial casting’s fracture morphology appeared extremely rough and irregular, with SEM revealing brittle features [48] (intergranular cleavage and river-like patterns), consistent with premature failure in the tensile test. In contrast, optimized castings exhibited fracture surfaces featuring abundant, uniformly distributed dimples—characteristic of ductile fracture with significant plastic deformation [49]. Notably, many dimples contained spherical particles at their bases, indicating particle-induced microvoid coalescence inclusions/second-phase particles nucleate microvoids that grow and coalesce during deformation. This ductile mechanism explains the optimized castings’ superior *σ*_b_ through efficient energy absorption and crack propagation resistance [50], confirming the efficacy of the self-evolving discovery through refined fracture morphologies.

Besides the above SEM observation after failure, we also performed EDS analysis on raw castings after demolding, obtaining oxygen elemental maps (Fig. 5d). Herein, specimen preparation involved sectioning cast rods (top/middle/bottom) via low-speed cutting and polishing. Compared to optimized castings, the initial casting showed markedly higher oxygen content, attributable to excessive gas generation and incomplete gas release during casting, consistent with the subpar GG/AP performances of the initial mold. Elevated oxygen concentrations indicate increased oxides/reaction residues, reducing material homogeneity and impairing local bonding quality [51], ultimately explaining the poor *σ*_b_ of the initial casting.

## CONCLUSION

In summary, we developed a self-evolving experimental platform, Ex3DSP, for casting performance optimization of sand molds. By integrating a robotic body (two functional islands comprising eight self-developed/modified automation workstations for standardized experimental data generation to achieve ‘can-do’ capability) with a multi-objective active learning brain (directionally tuning experimental protocols to ensure ‘can-think’ capability), the Ex3DSP constructed a ‘do-while-thinking’ self-evolving experimentation paradigm. For pedagogical demonstration, the Ex3DSP was used to address a high-dimensional optimization problem for three key objectives (casting performances of sand molds, including air permeability, gas generation, and compressive strength) against three main variables (process parameters of 3D printing, including nozzle voltage, layer thickness of sand, and curing agent proportion in sand). The Ex3DSP successfully discovered the Pareto front after 60 combinations, achieving a 1148-fold reduction in experiment workload compared to exhaustive evaluation of 68 921 potential combinations. The Pareto front suggested two types of optimal solutions (objective-optimal type with their dominant performance maximized at the expense of other performances, and balanced type across all performances) with interpretability (exploratory search, backbone construction, convergence filtering, and marginal exploitation). Interestingly, variable-objective interaction mechanisms (e.g. dominance of the ratio from nozzle voltage to curing agent proportion) and sand mold fracture mechanisms (e.g. strength-embrittlement trade-off caused by adhesive and cohesive modes) were elucidated. Moreover, the optimal solutions were adopted to guide process parameter selection for real-world casting applications, achieving castings with enhanced physicochemical and mechanical performances (e.g. a 385% relative improvement in tensile strength). This study reports the Ex3DSP as a practical demonstration of a self-evolving experimentation paradigm for 3D sand printing, offering a powerful route to accelerate performance optimization and mechanistic understanding in additive manufacturing, with potential applications in primary screening prior to industrial intensification.

## METHODS

### Self-evolving experimental platform

Ex3DSP was established in a dedicated area of the laboratory, consisting of two functional islands comprising eight self-developed/modified automation workstations, two computers, and a multi-objective active learning framework, iteratively executing design-manufacture-characterize-learn cycles without human intervention to construct a ‘do-while-thinking’ self-evolving experimentation paradigm for 3D sand printing. The workstations integrate self-developed/modified devices with commercial units and are interconnected through unified interfaces, with the overall system architecture and communication scheme shown in Fig. S3. In parallel, a Python-based software system was developed to handle experimental scheduling, parameter control, data management, and result analysis, with the workflow detailed in Note S5 and Fig. S4.

### Objective functions and constraints

The objective functions and constraints can be described as:


(1) \begin{eqnarray*}
\max \left\{ \begin{array}{@{}l@{}} {{\mathrm{O}}}_1 = \frac{{{\mathrm{AP}}}}{{{\mathrm{A}}{{\mathrm{P}}}_{\mathrm{m}}}}\\
{{\mathrm{O}}}_2 = \frac{{{\mathrm{GG}}}}{{{\mathrm{G}}{{\mathrm{G}}}_{\mathrm{m}}}}\\
{{\mathrm{O}}}_3 = \frac{{{\mathrm{CS}}}}{{{\mathrm{C}}{{\mathrm{S}}}_{\mathrm{m}}}} \end{array} \right.,
\end{eqnarray*}

(2) \begin{eqnarray*}
{\mathrm{st}}\left\{ \begin{array}{@{}l@{}} {\mathrm{90 V < NV < 110 V}}\\
{\mathrm{200 }}\mu {\mathrm{m < LT < 400 }}\mu {\mathrm{m}}\\
{\mathrm{0}}{\mathrm{.3 wt\% < CAP < 0}}{\mathrm{.55 wt\% }} \end{array} \right..
\end{eqnarray*}

Here, the goal of optimization is to maximize objective functions O_1_, O_2_, and O_3_, subject to box constraints on three structural variables (NV, LT, and CAP).

### Testing procedure of the casting

Molten aluminum was poured into hollow cylindrical molds, cooled, and demolded to obtain aluminum rods. The rods were subsequently machined and polished to prepare tensile and smooth specimens, respectively. Tensile tests were conducted using a universal testing machine (Li Shi Instrument, China) with a maximum load capacity of 100 kN and a constant crosshead speed of 5 mm/min. After testing, the fracture surfaces of the tensile specimens were examined by SEM, while the surfaces of the smooth specimens were analyzed by EDS.

### Chemicals and materials

The binder was 200DPlus (SQ Group, China), and the curing agent was GC3D270 (SQ Group, China). The raw material was ceramic sand 650#, featuring a particle size distribution of 70/140 mesh.

## Supplementary Material

nwag352_Supplemental_File

## Data Availability

The data that support the findings of this study are available from the corresponding author upon reasonable request. All custom code used in this study was based on open-source Python packages, which are available from the corresponding author upon reasonable request.
