# Supplementary material for: Self-evolving experimental platform for 3D sand printing
Source: Natl Sci Rev. 2026 Jun 9;13(14):nwag352. doi: 10.1093/nsr/nwag352 (PMC13397575; doi:10.1093/nsr/nwag352)
Supplement: nwag352_Supplemental_File [file nwag352_supplemental_file.pdf]

Supplementary Information

Self-evolving experimental platform for 3D sand printing

Songtao Hu[1,*,†], Xiantong Zhang[1,†], Kaiming Tang[1,2], Haoran Li[1], Wenhui Lu[1], Zipeng Wang[1], Yinjun Deng[1], Bo Zhang[2], Xiaobao Cao[3,*], Xi Shi[1,*] and Zhike Peng[1,2,*]

[1]State Key Laboratory of Mechanical System and Vibration, School of Mechanical Engineering, Shanghai Jiao Tong University, Shanghai 200240, China；

[2]School of Mechanical Engineering, Ningxia University, Yinchuan 750021, China;

[3]Guangzhou National Laboratory, Guangzhou 510320, China

***Corresponding authors.** E-mails: hsttaotao@sjtu.edu.cn; cao_xiaobao@gzlab.ac.cn; xishi@sjtu.edu.cn; z.peng@sjtu.edu.cn

†Equally contributed to this work.

Note S1. Multi-objective active learning framework.

To efficiently optimize multiple objectives in sand mold 3D printing, an active learning framework was constructed that intertwines surrogate modeling with acquisition-guided experiment design. The surrogate model serves as an evolving proxy, capturing the latent relationships between variables and objectives. It not only yields mean predictions but also quantifies uncertainty, offering a sense of how confidently the model "believes" its own estimates. Drawing on this probabilistic insight, the acquisition function selectively pinpoints candidate conditions most likely to push the PF forward. Through such a feedback-driven cycle, the optimization becomes progressively self-refining rather than purely sequential. Within this scheme, the 3D printing process is parameterized by a set of controllable variables, while the resulting performance metrics - each reflecting a distinct aspect of process quality - are defined as follows:

$$\mathbf{x} = [x_1, x_2, x_3], \tag{1}$$

$$\mathrm{f}(\mathbf{x}) = [\,f_1(x),\, f_2(x),\, f_3(x)]. \tag{2}$$

where **x** denotes the variables - NV, LT, CAP and f(**x**) refers to the three objectives - AP, GG, and CS. The framework aims not merely to search the variable space, but to navigate it intelligently, uncovering the non-dominated combinations that form the PF. In doing so, it reveals the optimal trade-offs among the three performance goals.

The GPR, a Bayesian non-parametric model, was employed as the surrogate model to capture the probabilistic relationship between variables and objectives. By inferring a posterior distribution over functions consistent with the observed data, GPR provides both the mean prediction and uncertainty estimation, enabling data-efficient learning under limited and noisy experimental conditions. Each objective $f_i(\mathbf{x})$ was modeled independently using the modelListGP module in BoTorch, an open-source Bayesian optimization framework implemented on PyTorch. [1] The prior distribution of each model is expressed as:

$$f_i(\mathbf{x}) \sim \mathrm{GPR}\left(m_i(\mathbf{x}), k_i\left(\mathbf{x}, \mathbf{x}'\right)\right), \tag{3}$$

where $m_i(\mathbf{x})$ denotes the mean function (assumed constant), and $k_i(\mathbf{x}, \mathbf{x}')$, represents the covariance kernel describing the correlation between two process settings **x** and **x**′. A squared-exponential (radial basis function, RBF) kernel was adopted to model the smooth relationship between variables and objectives, defined as:

$$k_i\left(\mathbf{x}, \mathbf{x}'\right) = \sigma_{f,i}^2 \exp\left(-\frac{\left\|\mathbf{x}-\mathbf{x}'\right\|^2}{2l_i^2}\right). \tag{4}$$

where $\sigma^2_{f,i}$ is the signal variance controlling the amplitude of modeled fluctuations, and l_i denotes the characteristic length scale governing surface smoothness. Given a training dataset:

$$\mathcal{D} = \left\{\left(\mathbf{x}_j, \mathbf{y}_j\right)\right\}_{j=1}^{3}, \tag{5}$$

$$\mathbf{y}_j = [y_{j1}, y_{j2}, y_{j3}], \tag{6}$$

where each $\mathbf{x}_j$ represents one experimental condition and $\mathbf{y}_j$ the corresponding observed objectives, the posterior distribution of each objective at an unseen point x_* follows a GPR distribution:

$$f(\mathbf{x}_* | \; \mathcal{D}) \sim \mathrm{GPR}\left(m_{\mathrm{post}}(\mathbf{x}), k_{\mathrm{post}}\left(\mathbf{x}, \mathbf{x}'\right)\right). \tag{7}$$

The EHVI was adopted as the acquisition function to guide the selection of candidate combinations most likely to expand the PF. In multi-objective optimization, the hypervolume

metric serves as a quantitative measure of the portion of the objective space dominated by the PF, relative to a predefined reference point. It is mathematically defined as:

$$\mathrm{HV}(\mathcal{P}) = \mathrm{Volume}\left(\bigcup_{\mathbf{y}\in\mathcal{P}}\{\mathbf{z} \mid \mathbf{r}\prec\mathbf{y}\prec\mathbf{z}\}\right), \tag{8}$$

where P denotes the PF, and the symbol $\mathbf{r} < \mathbf{y} < \mathbf{z}$ represents the set of points $\mathbf{z}$ that are dominated by the Pareto-optimal solution $\mathbf{y}$ but dominate the reference point $\mathbf{r}$. Here, $\mathbf{r}$ is the reference point in the objective space, defined as the worst possible performance among all objectives (typically set to [0,0,0] after normalization). Each $\mathbf{y} = [y_1, y_2, y_3]$ corresponds to one Pareto-optimal solution, and $\mathbf{z} = [z_1, z_2, z_3]$ represents any feasible objective vector within the dominated region of P. To identify the most informative next candidate, the expected hypervolume improvement is defined as the expected gain in the dominated volume if a new candidate $\mathbf{x}$ was to be evaluated, given by:

$$\mathrm{EHVI}(\mathbf{x}) = E[\mathrm{H}\left(\mathcal{P}\cup\{\mathbf{y}^*\}\right) - \mathrm{H}(\mathcal{P})], \tag{9}$$

where $\mathbf{y}^* = f(\mathbf{x})$ is the predicted objective vector corresponding to candidate $\mathbf{x}$ as estimated by the trained GPR. This expectation is taken over the joint posterior distribution of $f(\mathbf{x})$, reflecting both the predicted mean and uncertainty of each objective. Finally, the candidate expected to yield the greatest expansion of the Pareto front is selected for experimental validation, formulated as:

$$\mathbf{x}_{\mathrm{next}} = \arg\max_{\mathrm{x}} \mathrm{EHVI}(\mathbf{x}). \tag{10}$$

The optimization of objectives was implemented using the optimize_acqf function from BoTorch, which can provide diverse local maxims by multiple restarts of a local optimization algorithm (L-BFGS-B) from differing initial points. Herein, the number of initial points was set to 10, with 5 restarts and candidate numbers $q = 30$. Additionally, to further balance exploitation and exploration, an epsilon-greedy strategy was applied as: ranking all the candidates, selecting the optimal point with 90% probability, or randomly choosing from the lower 30% quantile with 10% probability.

Moreover, the shared portion of the hypervolume denotes the region of objective space jointly occupied by multiple PF solutions, reflecting their overlapping contributions to the overall performance landscape.

Note S2. Comparison of different algorithms through in silico virtual experiments.

Construction and validation of response surface

The response surface was constructed via a regression on all existing experimental data (60 combinations) using a radial basis function (RBF) model. Given an input set of variable combinations (i.e., NV, LT, CAP), the response surface outputs predictions for the objectives (i.e., AP, GG and CS) as substitutes for experimental measurements. To evaluate the model, a leave-one-out cross-validation (LOOCV) was performed. The data points are evenly and symmetrically distributed about the 45° isocline, confirming the model validity.

In silico virtual experiments

For algorithm comparison, hypervolume was selected as the evaluation metric. As a widely used performance indicator in multi-objective optimization, hypervolume simultaneously reflects convergence quality and Pareto-front diversity within a unified measure, making it especially suitable for assessing overall optimization effectiveness in the present small-sample, three-objective setting. In silico virtual experiments were then conducted to evaluate optimization performance. To ensure repeatability, each in silico virtual experiment was repeated three times.

Results

For comparison, the hypervolume values have been renormalized using the real experimental data as the baseline. The compared algorithms showed different convergence behaviors, with some converging faster than the RBF and others more slowly. This outcome demonstrates that, for the present small-sample, three-objective, three-variable 3D sand-printing optimization problem, the RBF-kernel GPR surrogate model used in the experiment represents an appropriate and reliable modeling choice.

Note S3. Mechanistic and physical interpretations of variable-objective interactions.
The mechanistic/physical interpretations of the variable-objective interactions are provided below:

NV

NV shows a negative relation with AP, as a higher NV tends to increase binder droplet volume and local saturation, thereby blocking more pore channels, which aligns with observations in *Addit. Manuf.* 56, 102929. NV shows a negative correlation with GG, because a higher NV likely introduces more binder-derived organics and raises the actual gas-evolution tendency, which aligns with observations in *Rapid Prototyp. J.* 26, 309-318. In contrast, NV shows a positive correlation with CS, since larger droplet volume and higher local saturation can enhance binder bridge formation and interparticle bonding, also supported by observations in *Addit. Manuf.* 56, 102929.

LT

LT shows a positive correlation with AP, as a larger layer thickness tends to produce a relatively looser and more open pore structure, facilitating gas passage, which aligns with observations in *Rapid Prototyp. J.* 26, 309-318. LT shows a positive correlation with GG, since a more open structure associated with greater layer thickness may promote gas release, which aligns with observations in *Addit. Manuf.* 73, 103690 and *China Foundry* 20, 553-562. In contrast, LT shows a negative correlation with CS, because increased layer thickness can weaken interlayer bonding continuity and reduce local compactness, also supported by observations in *Addit. Manuf.* 73, 103690.

CAP

CAP shows a positive correlation with AP, as higher curing-agent conditions may alter bridge morphology and increase pore openness during curing, which aligns with observations in *Rapid Prototyp. J.* 26, 309-318. CAP shows a negative correlation with GG, because higher CAP can introduce more curing-related organic species and form additional thermally decomposable bonded structures, thereby increasing the actual gas-evolution tendency, which aligns with observations in *J. Manuf. Process.* 30, 313-319 and *Addit. Manuf.* 47, 102427. Similarly, CAP shows a negative correlation with CS, as excessive curing can compromise the mechanical integrity of the bonded structure, also supported by observations in *J. Manuf. Process.* 30, 313-319.

Note S4. Interpretability analysis based on SHAP and GAM.

To interpret how variables affect multiple objectives, an integrated framework combining SHAP and GAM was employed. This two-stage interpretability scheme first quantifies each variable's contribution to the objectives and then unveils their nonlinear interactions with the response space. Through this joint analysis, the intrinsic mechanisms captured by the Ex3DSP optimization framework become more transparent, bridging the model's learned knowledge with interpretable physical insights. SHAP, derived from cooperative game theory, assigns each variable a contribution value representing its marginal influence on the model output. For a predictive model $f(\mathbf{x})$ with a feature set N, the Shapley value ϕ_j for variable j is defined as the expectation of its marginal contribution across all possible feature subsets $S \subseteq N \setminus \{j\}$:

$$\phi_j = \sum_{S \subseteq N \setminus \{j\}} \frac{|S|!(|N|-|S|-1)!}{|N|!}[f(S \cup \{j\}) - f(S)]. \tag{11}$$

Here, $f(S)$ denotes the model prediction using only the subset S. This definition satisfies Shapley's axioms of efficiency, symmetry, additivity, and null contribution, ensuring that contributions are distributed fairly among all variables. According to the efficiency property, the model prediction for an individual sample i can be decomposed into the global mean value ϕ_0 and the sum of all feature contributions:

$$f(i) = \phi_0 + \sum_{j=1}^{|N|} \phi_{i,j}. \tag{12}$$

For a dataset containing M evaluated samples and $|N| = 3$ variables (NV, LT, and CAP), the mean absolute contribution of each variable x_i is given by:

$$C_i = \frac{1}{M}\sum_{j=1}^{M} \left|\phi_i^{(j)}\right|, \tag{13}$$

and its normalized importance is expressed as:

$$\tilde{C}_i = \frac{C_i}{\sum_{k=1}^{|N|} C_k} \times 100\%. \tag{14}$$

The normalized values $\tilde{C}_i$ thus represent the percentage contribution of each variable to the model output, forming the quantitative foundation for subsequent nonlinear fitting analysis.

To further investigate the nonlinear relationships between SHAP values and variables, a GAM was constructed. In this formulation, SHAP values serve as the dependent variable, and the normalized variables act as predictors. This approach enables the identification of monotonic or nonlinear trends without assuming any fixed parametric form. The additive model is expressed as:

$$g(E[y]) = \beta_0 + \sum_{i=1}^{p} f_i(x_i), \tag{15}$$

where y denotes the SHAP value for a given objective, $g(\cdot)$ is the link function (identity in this study), β_0 is the global intercept, and $f_i(x_i)$ is a smooth, nonparametric function describing the partial dependence of y on variable x_i. Each smooth term $f_i(x_i)$ is represented as a linear combination of basis functions $b_{ik}(x_i)$ weighted by coefficients β_{ik}:

$$f_i(x_i) = \sum_{k=1}^{K} \beta_{ik} b_{ik}(x_i), \tag{16}$$

where the basis functions (e.g., cubic splines or thin-plate regression splines) ensure adequate smoothness while preserving flexibility. Model parameters are obtained by minimizing a penalized least squares criterion that balances goodness of fit and smoothness:

$$\text{minimize}\left\| y-\beta_0-\sum_{i=1}^{p} f_i(x_i)\right\|^2+\sum_{i=1}^{p}\lambda_i\int\left[f_i^{''}(x_i)\right]^2 \mathrm{d}x_i, \tag{17}$$

where λ_i is the smoothing parameter controlling the trade-off between model flexibility and overfitting. The optimal λ_i values are determined using generalized cross-validation, ensuring that each smooth function $f_i(x_i)$ accurately reflects the intrinsic variable - SHAP dependency rather than noise-driven artifacts. The resulting fitted functions provide a continuous and interpretable mapping from each process variable to its SHAP value, enabling a mechanistic understanding of variable-objective interactions learned during the Ex3DSP self-evolving optimization process.

Note S5. Detailed workflow and implementation of the Ex3DSP platform.

The detailed workflow of Ex3DSP is provided below:

Variable design

The variable design method was employed to optimize the process parameters of the sand mold 3D printing workstation. A multi-objective active learning framework was subsequently applied to generate variable combinations of NV, LT, and CAP. The resulting sets were transmitted to the printing execution software and simultaneously uploaded to the printing server, where the updated parameters were implemented and the corresponding printing tasks executed.

Specimen manufacture

The main program first transmitted the updated process parameters and printing tasks to computer 1. In the material mixing workstation, raw sand and curing agent were blended at the prescribed ratio and discharged into a loading basket, which was subsequently transported by an AGV. The specimens were then fabricated using the self-developed 3D printing workstation. For each parameter combination, three AP specimens (π×25×25×50 mm^3), five GG specimens (π×5×5×10 mm^3), and three CS specimens (π×10×10×20 mm^3) were printed. Upon completion, a six-axis robotic arm transferred the specimens onto trays mounted in the designated post-processing area of the AGV for transportation.

Specimen post-processing

Post-processing is essential to remove residual loose sand adhering to the specimen surface, thereby preventing interference during subsequent characterization. The specimens were first transported by the AGV to the post-processing area and then transferred by a six-axis robotic arm to the cleaning workstation, where surface treatment was performed using an air-blow module (AIKER, China) at an airflow velocity of 82 m/s for 5 minutes. Following cleaning, the GG specimens were weighed at the weighing workstation and placed on the AGV tray within the testing section, whereas the remaining specimens were directly positioned in their respective testing zones.

Specimen characterizing

Before characterization, the AGV moved to the characterization island and performed visual positioning, after which each specimen was sequentially picked and tested. The AP was measured first using a Molding Air Permeability Tester (San Feng, China) and calculated according to the following equation.

$$\mathrm{AP} = \frac{QH}{PFt}, \tag{18}$$

where Q is the gas volume (mL) passing through the specimen, H is the specimen height (50 mm), P is the applied gas pressure (Pa), F is the cross-sectional area of the specimen ($F = \pi r^2$, r = 25 mm) and t is the time (s) required for the gas to pass through the specimen under constant pressure. GG tests were performed using an Intelligent Molding Gas Generation Tester (San Feng, China). Each specimen was heated in a furnace at 850°C for 1 min, during which the evolved gas volume was measured. The GG value was subsequently calculated using the following equation:

$$\mathrm{GG} = \frac{1000}{L_1 - L_0}, \tag{19}$$

where L_0 is the initial reading (mL/g), L_1 is the final reading (mL/g), and L_1–L_0 represents the gas volume released per unit mass of specimen. The coefficient 1000 is used for unit conversion, so

that the GG is expressed in g/L. A quasi-static uniaxial compression test was conducted using a universal testing machine (Tian Yuan Test Instrument, China) within the compression test workstation, with a load capacity of 5 kN and a resolution of 0.03%. The test was performed at a constant crosshead speed of 8 mm/min and terminated when either 60% strain or the maximum load capacity was reached. Upon completion, the specimen was automatically removed from the test platen by a sweeping module. The CS was then calculated as:

$$\mathrm{CS} = \frac{F_c}{\pi r^2}, \tag{20}$$

where F_c represents the failure load, and r = 10 mm denotes the radius of the specimen cross-section. The F_c value was automatically extracted from the force-displacement curves using a Python-based data analysis pipeline. Both the raw data generated during the characterization stage and the processed objectives (e.g., AP, GG, and CS) were stored in a local database on computer 2, serving as the labeled dataset for model training.

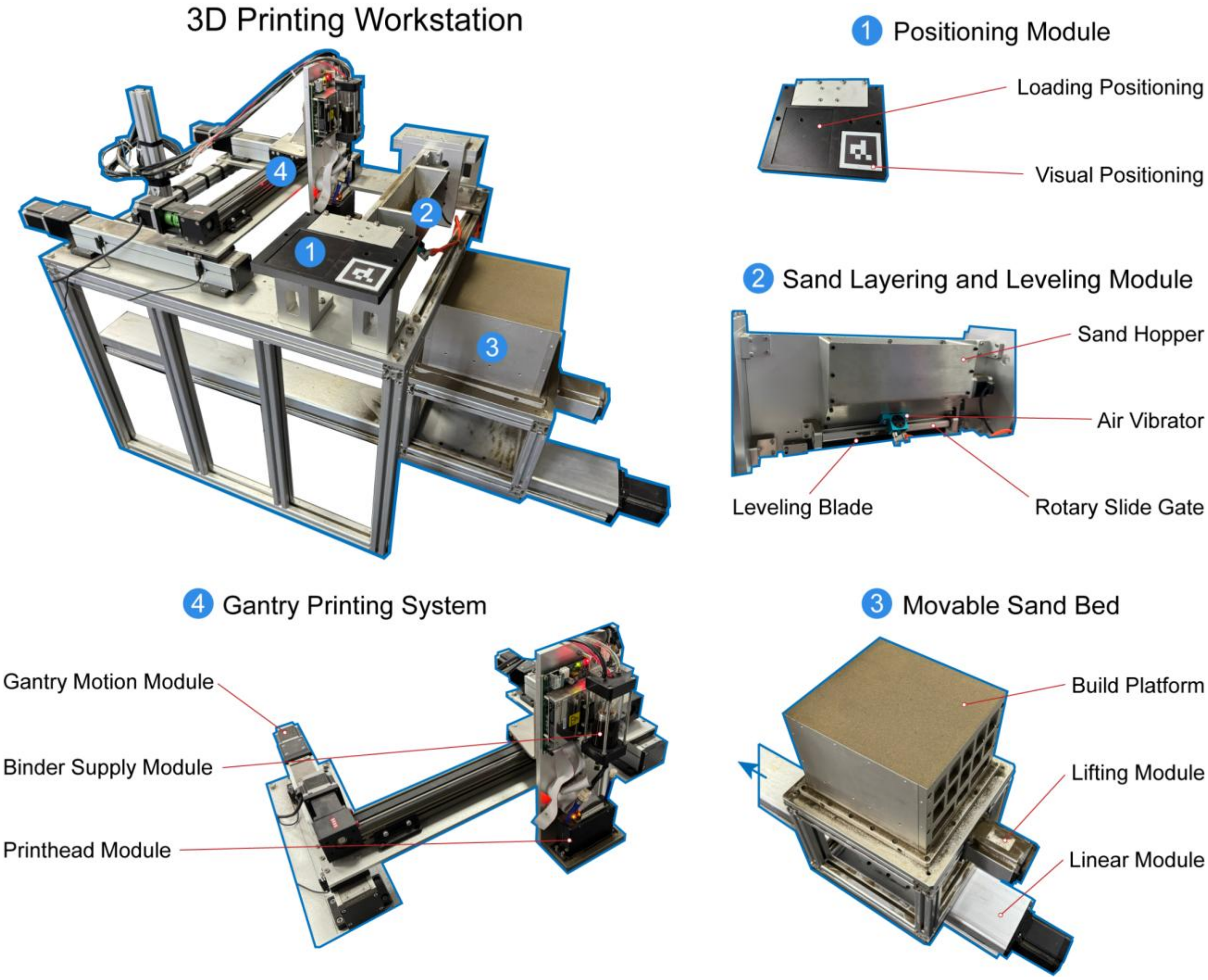

Fig. S1. Self-developed 3D printing workstation of the Ex3DSP platform.

The 3D printing workstation consists of four self-designed modules for automated sand mold fabrication: a positioning module, a sand layering and leveling module, a movable sand bed, and a gantry printing system. Working synergistically, these modules execute sand spreading, leveling, and binder jetting within a unified mechanical framework. The positioning module performs both visual and mechanical alignment during sand box loading. The sand layering and leveling module manages sand feeding through a vibratory hopper and rotary slide gate. The movable sand bed provides precise vertical motion and planar positioning, while the gantry printing system integrates the printhead, binder supply, and motion control modules to achieve high-precision binder deposition. Together, the four modules enable fully automated, repeatable, and programmable sand mold printing within the Ex3DSP platform.

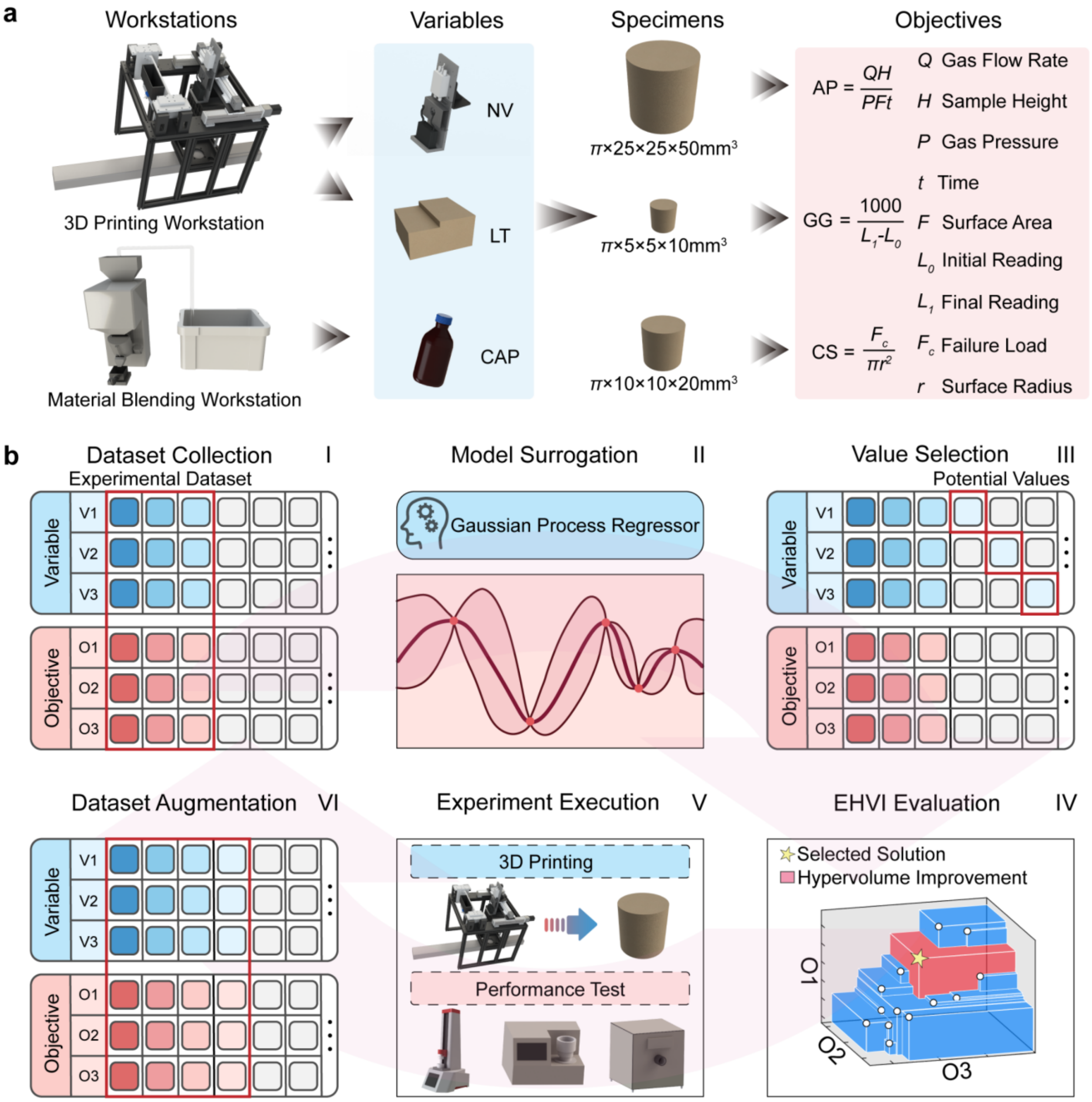

Fig. S2. Multi-objective high-dimensional optimization.

(a) High-dimensional optimization problem with 3 objectives and 3 variables. **(b)** Multi-objective active learning framework providing self-evolving solutions.

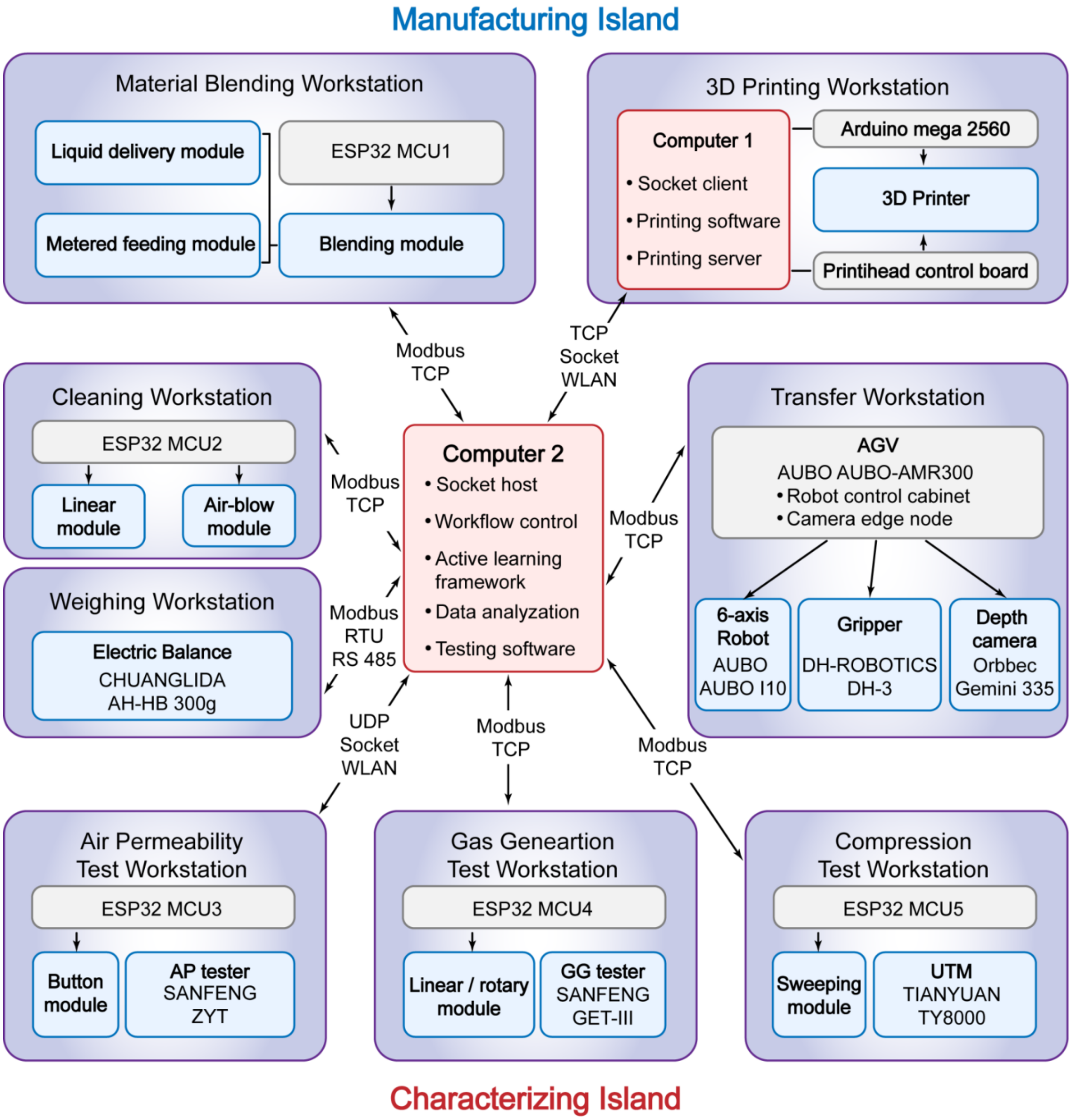

Fig. S3. Organization of the self-evolving experimental platform (Ex3DSP).

Include the configuration of workstations, device composition, communication architecture, and software deployment. Purple boxes denote the workstations; blue boxes indicate the devices or functional modules within each workstation; grey boxes represent the control and communication terminals; and red boxes correspond to the two computers responsible for system-wide control and management.

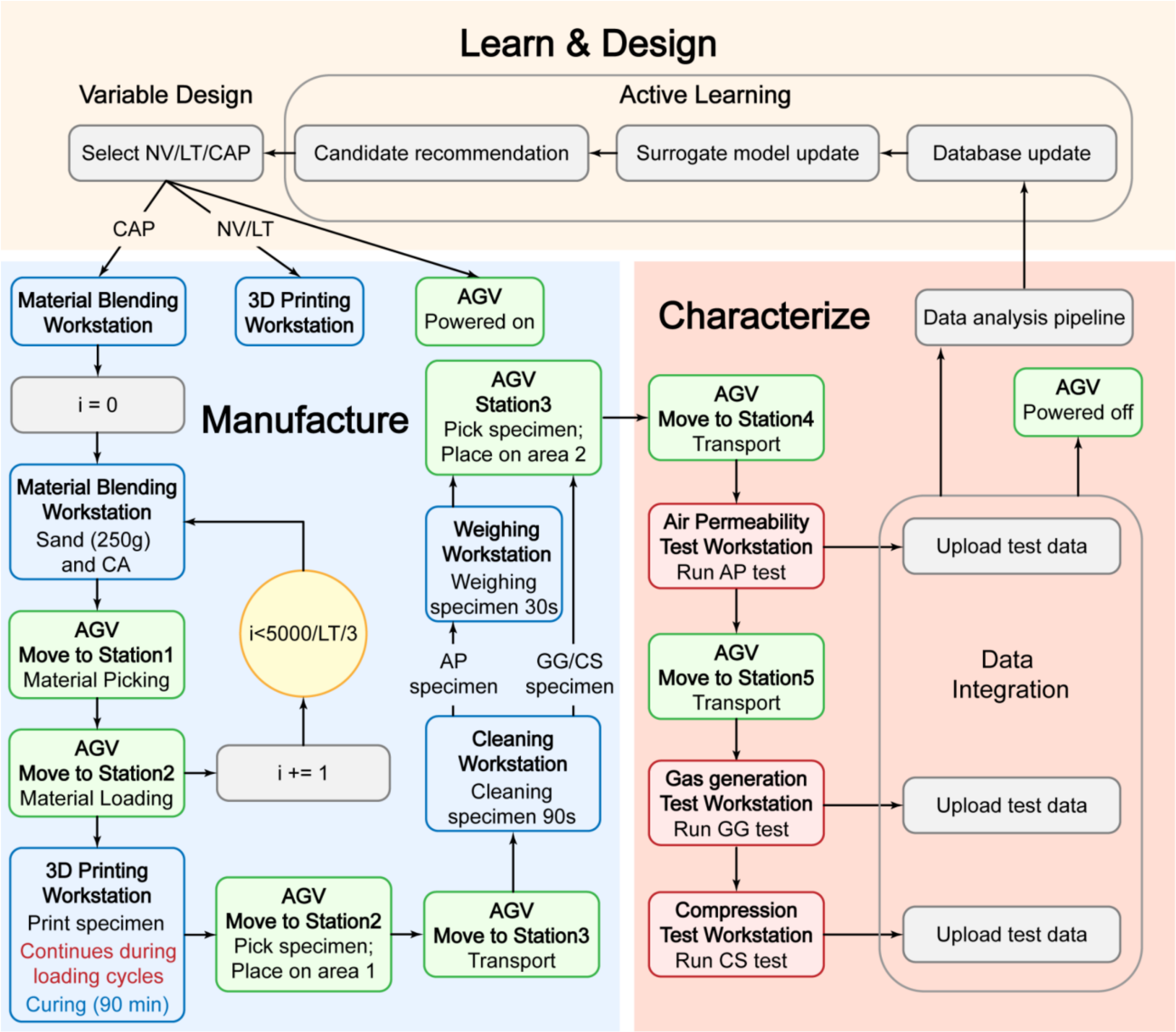

Fig. S4. Detailed workflows and corresponding execution workstations for each step.
The main control software orchestrates the entire design-manufacture-characterize-learn cycle within a closed-loop framework, enabling automated coordination of all experimental operations. Through multithreaded execution, specimens fabricated by multiple printing workstations are processed in parallel, ensuring standardized procedures and consistent data generation across the system.

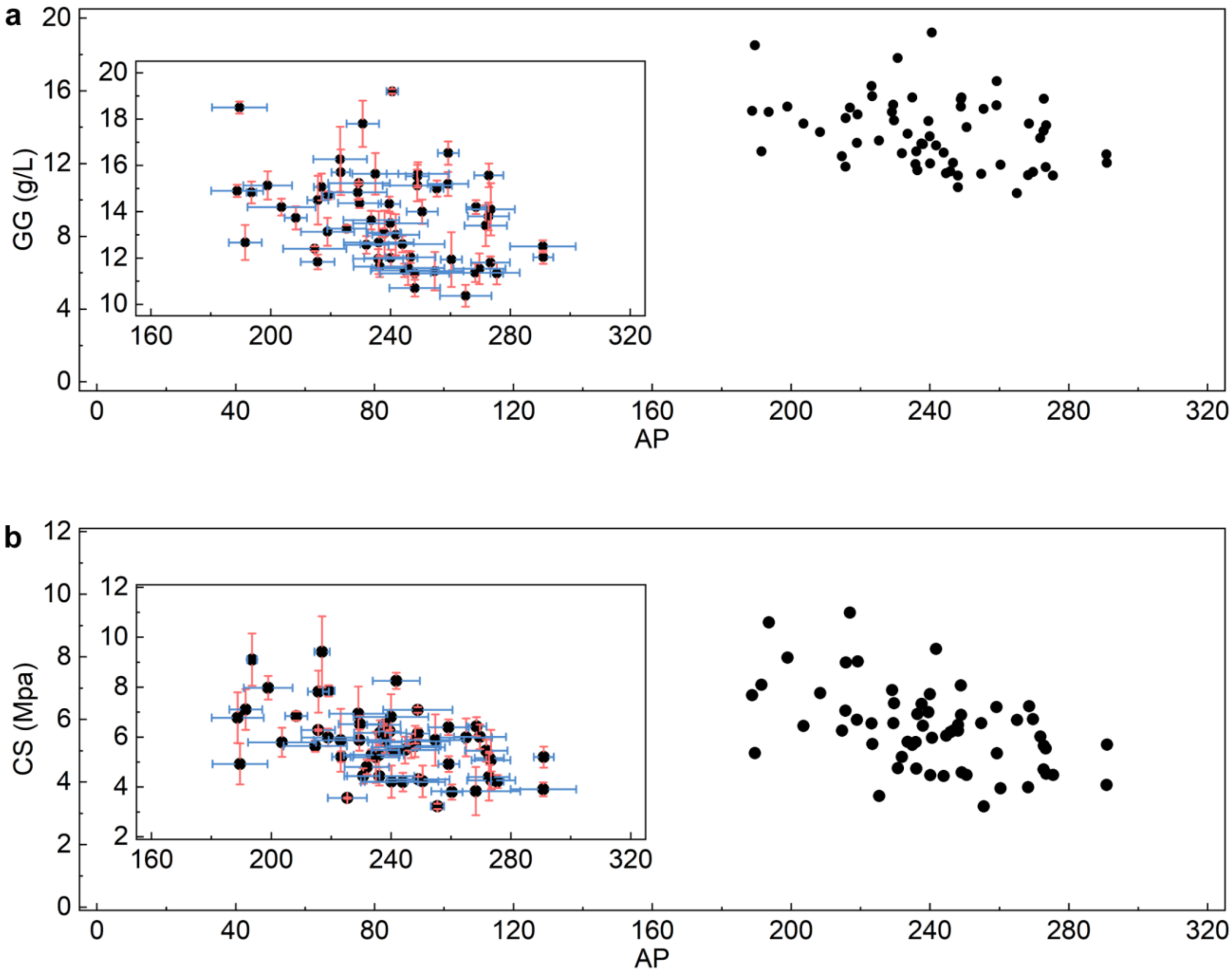

Fig. S5. Objective performance with standard deviations obtained from the dataset generated by the self-evolving experimental framework.

(a) Comparison between AP and GG. **(b)** Comparison between AP and CS. It should be noted that the relatively large deviation observed in AP is considered normal, which originates from the stochastic nature of sand particle packing and the inherent randomness of pore connectivity in the printed specimens.

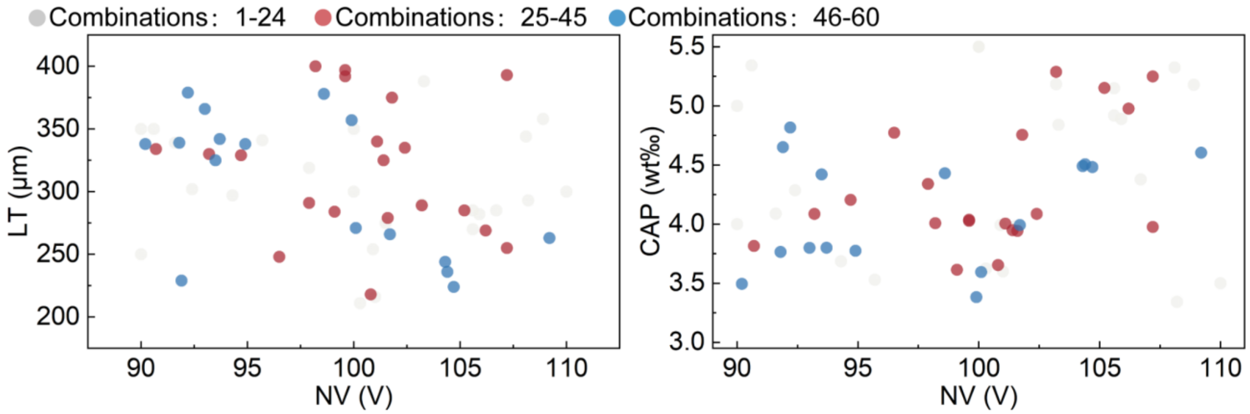

Fig. S6. Side views of the variable space across different cycles.

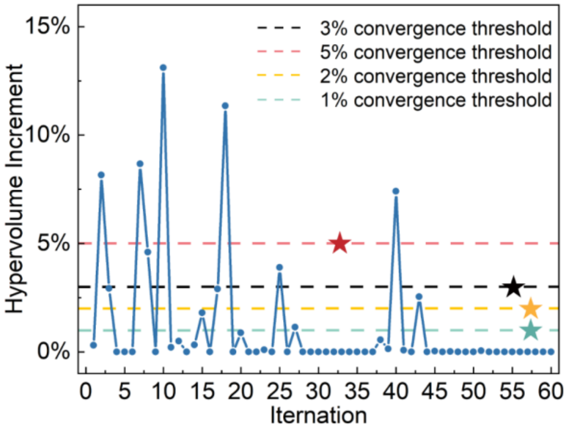

Fig. S7. Iteration-by-iteration hypervolume increment.

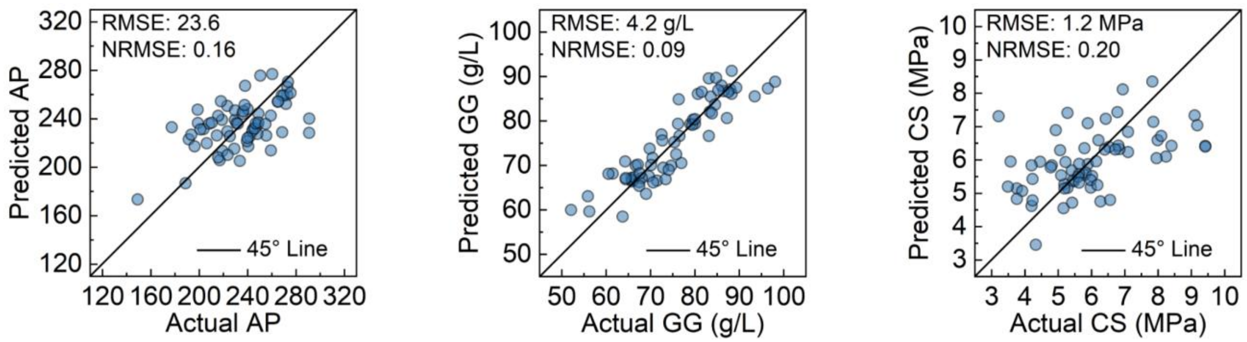

Fig. S8. LOOCV on the constructed response surface. (a) Prediction on AP. **(b)** Prediction on GG. **(c)** Prediction on CS.

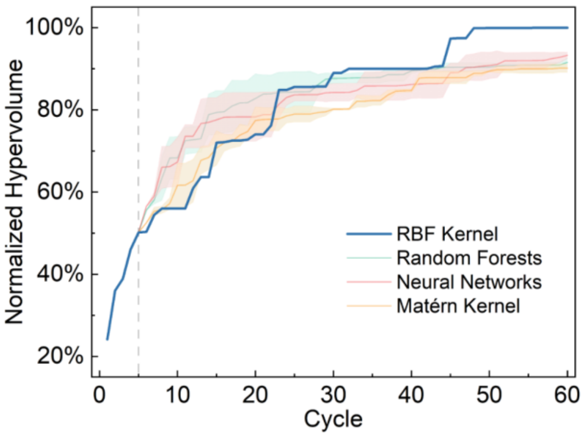

Fig. S9. Evolution of hypervolume over iterations from the in silico virtual experiments.

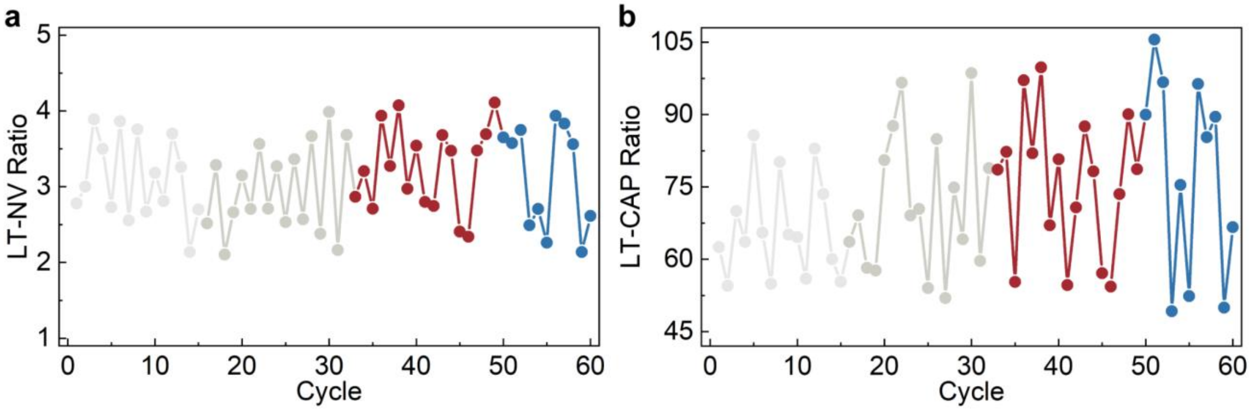

Fig. S10. Results of variable ratios.

(a) LT-NV ratio. **(b)** LT-CAP ratio. LT is at a different physicochemical level from NV and CAP, and therefore shows no clear or consistent trend with sand mold performance.

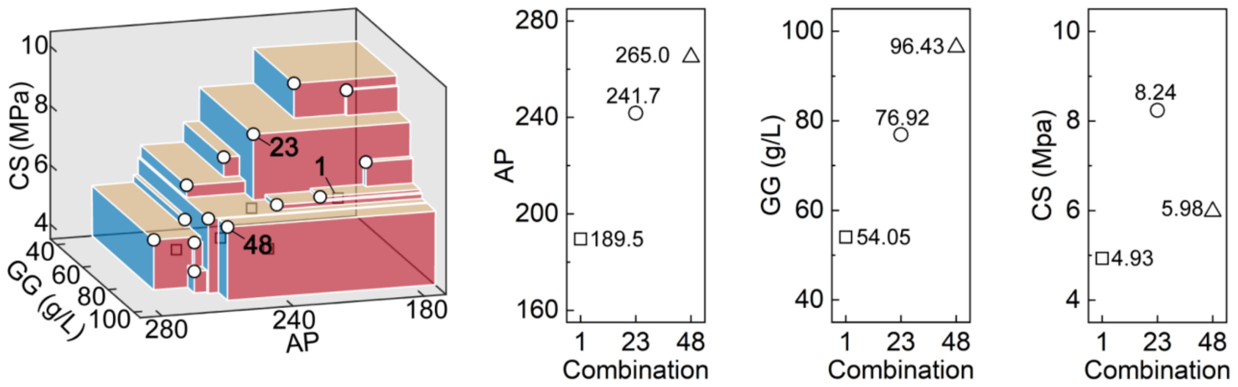

Fig. S11. Two optimized combinations selected from the discovered Pareto front to guide casting, with the initial combination as reference.

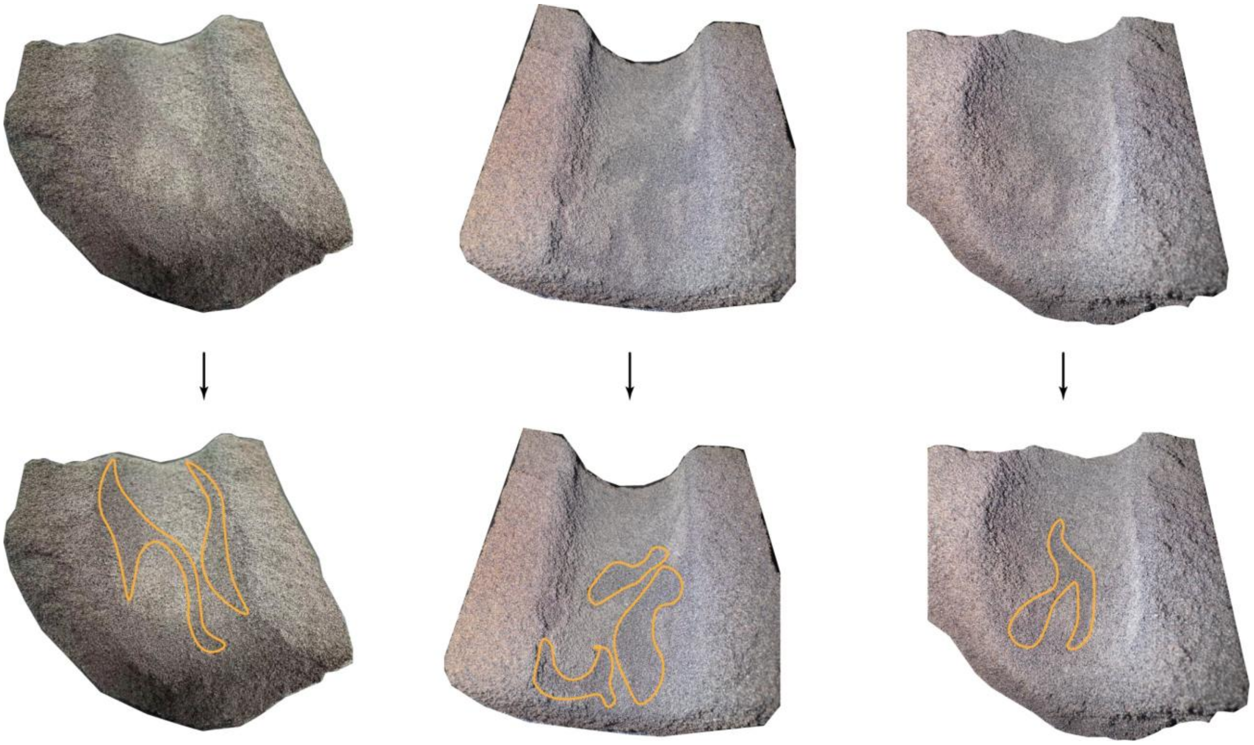

Fig. S12. Fragments of the mold from Combination 1 after casting.

During the casting process, delamination occurred due to insufficient mechanical strength of the sand mold (in yellow).

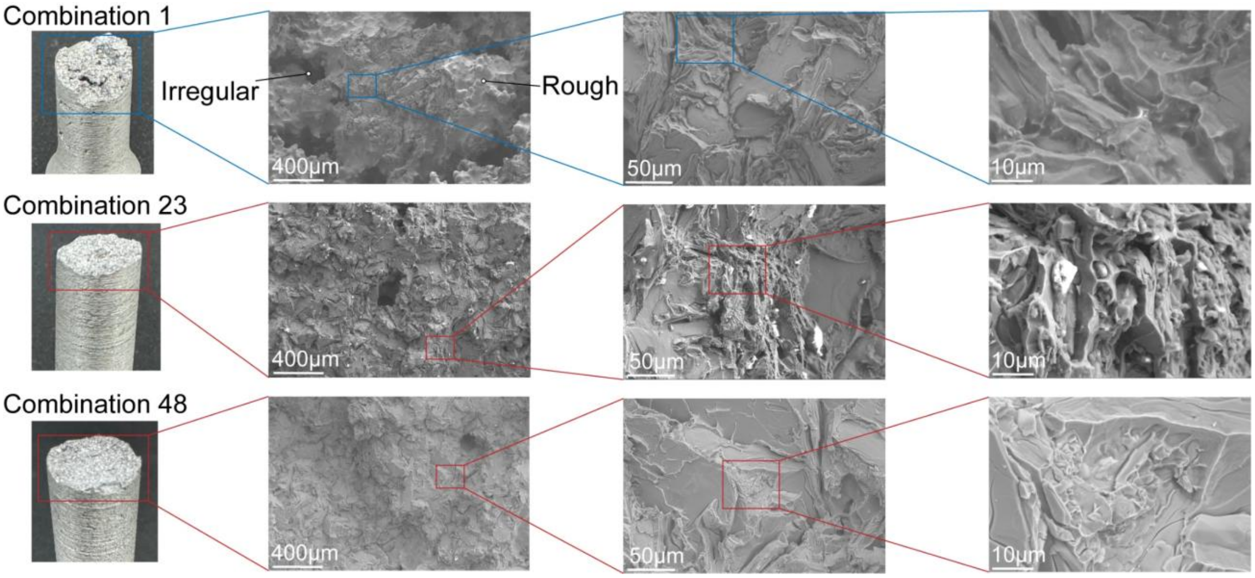

Fig. S13. Fracture morphologies of tensile specimens observed by SEM at three magnifications.

The images compare the reference group (Combination 1) and the optimized groups (Combinations 23 and 48).

Table S1. Variables affecting the most critical performances of printed sand molds.

Performance	Variable	Reason
AP	NV<br>LT<br>CAP<br>binder composition<br>curing-agent composition<br>reclaimed sand ratio<br>sand composition<br>sand morphology	These variables jointly determine droplet infiltration behavior, particle packing state, and pore-channel connectivity.
GG	NV<br>LT<br>CAP<br>binder composition<br>curing-agent composition<br>reclaimed sand ratio<br>sand composition	These variables govern the distribution and reaction state of the binder/curing system, as well as the amount and release pathway of gaseous products during heating.
CS	NV<br>LT<br>CAP<br>binder composition<br>curing-agent composition<br>reclaimed sand ratio<br>sand morphology	These variables regulate droplet deposition quality, bridge formation, interparticle bonding, and microstructural integrity.

Summary of variables affecting the most critical performance indicators of printed sand molds (AP, GG, and CS). Six variables (i.e., NV, LT, CAP, binder composition, curing-agent composition, and reclaimed sand ratio) could simultaneously influence AP, GG, and CS. Considering practical operability within the current platform, we selected NV, LT, and CAP, eventually.

In future work, the remaining variables (e.g., binder composition, curing-agent composition, and reclaimed sand ratio) can be progressively incorporated considering the platform. For example, binder composition and curing-agent composition could be integrated through automated formulation, metering, and dispensing modules, while reclaimed sand ratio could be addressed via automated sand recovery, proportioning, and feeding modules. Such extensions would further expand variable space and enhance platform capability.

Table S2. Perturbation validation results for the representative Pareto solutions.

Case	NV (V)	LT (μm)	CAP (wt‰)	AP	GG (g/L)	CS (MPa)
**Combination 23 (Balanced)**						
**Original**	**101.5**	**275**	**3.98**	**241.7**	**76.92**	**8.24**
Perturbation 1	102	280	3.93	223.53	75.76	7.96
Perturbation 2	102	270	3.93	240	75.36	8.09
Perturbation 3	101	280	4.03	230.13	80.59	7.17
Perturbation 4	101	270	4.03	235.23	72.64	7.54
**Combination 45 (CS-optimal)**						
Case	NV (V)	LT (μm)	CAP (wt‰)	AP	GG (g/L)	CS (MPa)
**Original**	**109.2**	**263**	**4.60**	**216.90**	**66.36**	**9.42**
Perturbation 1	109.7	268	4.55	196.22	67.57	9.18
Perturbation 2	109.7	258	4.55	216.53	64.38	9.41
Perturbation 3	108.8	268	4.65	232.12	66.67	8.1
Perturbation 4	108.8	258	4.65	206.20	72.2	8.4

Table S3. Variable combinations and objective performance in the dataset.

Iternation	Combination			Objective Performance			Pareto Front
	NV (V)	LT (μm)	CAP (wt‰)	AP	GG (g/L)	CS (MPa)	
0	90	250	4.00	189.5	54.05	4.93	N
0	100	300	5.50	249.1	63.98	4.30	N
0	90	350	5.00	234.9	63.98	5.22	N
0	100	350	5.50	273.4	70.92	4.30	N
0	110	300	3.50	236	78.93	4.42	N
1	90.6	350	5.34	272.7	72.46	4.33	N
2	105.6	270	4.92	229.4	65.66	5.89	N
3	103.3	388	4.84	237.9	76.51	5.79	N
4	106.7	285	4.38	255.4	66.67	3.21	N
5	108.1	344	5.32	250.5	71.43	4.23	N
6	103.2	290	5.18	223.1	61.46	5.89	N
7	91.6	339	4.09	268.2	87.95	3.76	N
8	97.9	319	4.34	290.8	80.00	3.92	N
9	101	216	3.60	230.7	56.18	4.46	N
10	105.6	285	5.15	248.9	66.09	7.10	Y
11	100.9	254	3.99	259.2	60.50	4.93	N
12	108.9	358	5.18	272.8	64.27	5.16	N
13	100.3	211	3.62	240.5	52.08	5.41	N
14	105.9	282	4.89	229.6	69.59	6.53	N
15	94.3	297	3.69	191.4	78.93	7.10	Y
16	108.2	293	3.34	188.7	67.11	6.78	N
17	95.7	341	3.53	248	88.26	5.83	N
18	101.5	275	3.98	241.7	76.92	8.24	Y
19	92.4	302	4.29	231.9	79.55	4.81	N
20	106.2	269	4.98	268.5	70.57	6.43	Y
21	101.1	340	4.00	244.6	87.18	5.47	N
22	96.5	248	4.77	233.5	73.37	5.28	N
23	107.2	393	5.25	271.7	74.63	5.44	Y
24	107.2	255	3.98	198.9	65.79	7.99	N
25	99.6	397	4.03	248	93.46	5.63	N
26	100.8	218	3.65	223.3	63.69	5.22	N
27	101.8	375	4.76	290.9	83.13	5.19	Y
28	99.1	284	3.61	239.5	69.78	6.21	N
29	101.4	325	3.95	243.9	79.37	4.20	N
30	105.2	285	5.15	259.1	65.79	6.40	N
31	99.6	392	4.04	245.8	86.43	5.60	N
32	102.4	335	4.09	218.9	76.16	5.98	N
33	98.2	400	4.01	246.6	83.13	5.63	N
34	97.9	291	4.34	225.3	75.36	3.57	N
35	93.2	330	4.09	235.8	83.54	5.28	N

(continued on next page)

(continued from above page)

36	103.2	289	5.29	248.9	64.39	6.14	N
37	101.6	279	3.94	203.5	70.42	5.79	N
38	90.7	334	3.82	269.6	89.29	6.02	Y
39	94.7	329	4.21	215.6	84.53	6.27	Y
40	109.2	263	4.60	216.9	66.36	9.42	Y
41	104.3	244	4.49	193.5	67.43	9.10	Y
42	93.5	325	4.42	240	83.33	4.20	N
43	91.8	339	3.77	265	96.43	5.98	Y
44	92.2	379	4.82	260.3	83.82	3.76	N
45	93.7	342	3.80	236.3	85.98	6.18	Y
46	99.9	357	3.38	214.6	80.65	5.63	N
47	90.2	338	3.50	273.3	84.75	5.06	Y
48	91.9	229	4.65	219.1	68.03	7.86	N
49	100.1	271	3.59	208.3	72.83	6.84	N
50	104.4	236	4.51	229	67.43	6.94	N
51	93	366	3.80	275.4	88.26	4.23	Y
52	98.6	378	4.43	237.5	76.34	6.56	N
53	94.9	338	3.78	254.7	87.49	5.09	N
54	104.7	224	4.48	215.7	68.97	7.83	N
55	101.7	266	3.99	239.9	74.07	6.81	N

56	109.3	204	4.09	149.1	55.87	9.18	N
57	94.9	367	4.25	277.3	82.64	4.77	N
58	98.7	373	4.11	198.7	81.52	5.19	N
59	102.8	260	4.99	210.3	64.38	8.10	N
60	105.2	307	3.56	223.5	75.76	7.96	N

Table S4. Effect reversal of NV on CS (red: reversal interval; blue: responsible variable).

NV (V)	SHAP Value	LT (μm)	CAP (wt‰)
92.2	-0.041649	379	4.82
92.4	-0.013283	302	4.29
93	-0.161653	366	3.80
93.2	0.00567	330	4.09
93.5	0.08408	325	4.42
93.7	0.312985	342	3.80
94.3	0.389433	297	3.69
94.7	0.334602	329	4.21
94.9	0.349563	338	3.78
95.7	0.075929	341	3.53
96.5	-0.294066	248	4.77
97.9	-0.194693	319	4.34
97.9	-0.198595	291	4.34

Within this NV range, the SHAP value should be negative. However, due to coupling with CAP, an effect reversal occurs.

NV (V)	SHAP Value	LT (μm)	CAP (wt‰)
105.6	0.140354	270	4.92
105.6	0.192074	285	5.15
105.9	0.066398	282	4.89
106	-0.503749	350	3.44
106.2	-0.272294	269	4.98
106.7	-0.789497	285	4.38
107.2	-0.123828	393	5.25
107.2	0.272291	255	3.98
108.1	0.082434	344	5.32
108.2	0.445589	293	3.34

Within this NV range, the SHAP value should be positive. However, due to the excessively high LT values and the coupling with CAP, an effect reversal occurs.

Table S5. Effect reversal of CAP on CS (red: reversal interval; blue: responsible variable).

CAP (wt‰)	SHAP Value	NV (V)	LT (μm)
4.34	-0.783363	97.9	291
4.38	-0.589603	106.7	285
4.42	-0.512531	93.5	325
4.43	0.552392	98.6	378
4.48	0.786973	104.7	224
4.49	0.793464	104.3	244
4.51	0.793464	104.4	236
4.60	0.634049	109.2	263
4.65	0.691847	91.9	229
4.76	-0.099139	101.8	375
4.77	-0.132587	96.5	248
4.82	-0.034791	92.2	379

Within this CAP range, the SHAP value should be negative. However, due to the excessively low LT values and the coupling with NV, an effect reversal occurs.

Movie S1. Manufacture and characterization performed by Ex3DSP.

Reference

1. Balandat M, Karrer B, Jiang DR *et al*. BoTorch: A framework for efficient Monte-Carlo Bayesian optimization. *In: Proceedings of the 34th International Conference on Neural Information Processing Systems*. 2020, 21524.
